# Supplementary material for: Identification of Quantitative Trait Loci for Node Number, Pod Number, and Seed Number in Soybean
Source: Int J Mol Sci. 2025 Mar 5;26(5):2300. doi: 10.3390/ijms26052300 (PMC11900990; doi:10.3390/ijms26052300)
Supplement: Supplementary file 1 [file ijms-26-02300-s001.zip › ijms-3468356-supplementary.pdf]

Tables S1 Functional annotations of potential candidate genes.

ID	Description	GO annotation for biological progress
Glyma.19G191600	Serine/threonine-protein kinase CTR1	regulation of stem cell division;response to fructose;negative regulation of ethylene-activated signaling pathway;gibberellin biosynthetic process;protein serine/threonine kinase activity
Glyma.19G193100	Serine/threonine-protein kinase KIPK1	cytoplasm;nucleus;plasma membraneprotein kinase binding;protein serine/threonine kinase activity
Glyma.19G194800	Cell division protein FtsZ homolog 2-1	cytoplasm;chloroplast fission;chloroplast stroma;protein self-association;chloroplast thylakoid;GTPase activity; GTP binding;chloroplast thylakoid membrane; chloroplast
Glyma.19G195300	Kinesin-like protein KIN-5C	Cytoplasm;spindle;microtubule motor activity;microtubule-based movement;microtubule binding;microtubule;ATP binding
Glyma.19G196000	Probable UDP-N-acetylglucosamine--peptide N-acetylglucosaminyltransferase SPINDLY	negative regulation of gibberellic acid mediated signaling pathway;cytoplasm;;rhythmic process;regulation of reactive oxygen species metabolic process;flower development;gibberellic acid mediated signaling pathway;cytokinin-activated signaling pathway;protein glycosylation;cell differentiation;cytosol;nucleus
Glyma.19G196300	mRNA-decapping enzyme-like protein	mRNA binding;deadenylation-dependent decapping of nuclear-transcribed mRNA;enzyme activator activity;deadenylation-independent; decapping of nuclear-transcribed mRNA; Cytoplasm;mRNA processing;P-body;hydrolase activity;defense response
Glyma.19G199100	N-acetyltransferase	damaged DNA binding;DNA-directed DNA polymerases
Glyma.19G199400	IPR011333 - POZ domain	BTB/POZ domain-containing protein
Glyma.19G199900	obsolete mitochondrial alpha-ketoglutarate/malate transport	Aluminium activated malate transporter family protein
Glyma.19G200300	DOF ZINC FINGER PROTEIN DOF1.1-RELATED	obsolete regulation of DNA-templated transcription in response to stress
Glyma.19G200400	Protein with predicted involvement in meiosis	Tetratricopeptide repeat (TPR)-like superfamily protein
Glyma.19G200800	NUCLEAR TRANSCRIPTION FACTOR Y SUBUNIT A-10-RELATED	transcription factor activity, sequence-specific DNA binding; regulation of transcription, DNA-templated
Glyma.19G200900	Glutaredoxin and related proteins // Uncharacterized conserved protein	Glutaredoxin family protein
Glyma.19G201100	ubiquitin carboxyl-terminal hydrolase	ubiquitin-specific protease 8
Glyma.19G201200	folic acid-containing compound metabolic process	Dihydroneopterin aldolase
Glyma.19G201300	no description	no description
Glyma.19G201400	SF125 - SERINE/THREONINE-PROTEIN KINASE // SUBFAMILY NOT NAMED	calmodulin-domain protein kinase cdpk isoform 2

Table S2. Number distribution of high quality polymorphic SLAF on each chromosome

ChrID	SLAF Number	ChrID	SLAF Number
Gm01	349	Gm12	134
Gm02	388	Gm13	244
Gm03	366	Gm14	225
Gm04	310	Gm15	626
Gm05	184	Gm16	570
Gm06	212	Gm17	346
Gm07	372	Gm18	284
Gm08	256	Gm19	690
Gm09	358	Gm20	214
Gm10	457	Other	0
Gm11	132	Total	6,717

Table S3. Primer sequences of Quantitative real-time PCR

Gene	Forwerd	Reverse
Glyma.19G199100	CACCTTGGTTATCCTGCCCT	GAGGATGAATATTGTTGTGCCATGT
Glyma.19G199400	CACCTGTATGAGGAGCACGG	GGGGCAGATGGAATAGCCTC
Glyma.19G199900	CCCTACACTAAGAGGGTGGT	CGTAGGGCTCAGCAGGAAGT
Glyma.19G200300	CCACAAACTACTCTTCCACCCT	TCTTCTCCCCATTCGTGCTT
Glyma.19G200400	AAATTCCCTTCAGGCCCCAG	CCTCCACTGGGATTTTTGTGG
Glyma.19G200800	GCTTGTTACTTTCCTGCACCAC	GCATTGCAATTGTGGGCTGT
Glyma.19G200900	CTGTGTACTGATGACTCGGGA	GCAATCCCCAGCATGATCGTTA
Glyma.19G201100	TAAAGGCTGGTCAAGTGTGGC	AGTGAACCCTGGGACAAGTG
Glyma.19G201200	TCCCTTGTTGCCTTCTGACC	GGCTGCCAAAAGTCTTGAACC
Glyma.19G201300	ATGGTGCATCGCAGGACTAC	TGCATCCAGTACCACAGTGC
Glyma.19G201400	TACACAGTTGCTCCACTGCC	TTTTCGCGACATGAACACGG
Glyma.19G191600	CTATCACCGGCTTTGGCAGT	CACGAGAGACAGACAAGCAC
Glyma.19G193100	CACATAAGTAGCAGAAGCAGCC	TTTCTTCCAGCTCCACGGAC
Glyma.19G194800	CTGCCCCTCCTCCAGTTATC	TGGTCGGGGACAGAGAGATG
Glyma.19G195300	GACAGCTTTCTGTGGACCCT	CGTCAGTGTCCAGCTTTTGG
Glyma.19G196000	TCCTTCTCCAGTTGAACCCG	CTTTCACAGCGTTTTGCTGC
Glyma.19G196300	AGAGGGTGGTCCACTGCC	CACTGTGAACACAGTC
